# Hemodynamic Performance of a Self-Expanding Transcatheter Aortic Valve with an Intra-Annular Leaflet Position in Patients with a Small Aortic Annulus †

**DOI:** 10.3390/medicina61040661

**Published:** 2025-04-03

**Authors:** Matjaž Bunc, Gregor Verček, Ole De Backer

**Affiliations:** 1 Department of Cardiology, University Medical Centre Ljubljana, 1000 Ljubljana, Slovenia; 2 Faculty of Medicine, University of Ljubljana, 1000 Ljubljana, Slovenia; 3 Department of Cardiology, The Heart Centre, Rigshospitalet, Copenhagen University Hospital, 2100 Copenhagen, Denmark

**Keywords:** transcatheter aortic valve implantation, TAVI, TAVR, small aortic annulus, self-expanding, intra-annular, transcatheter aortic valve

## Abstract

*Background and Objectives*: Transcatheter aortic valve implantation is associated with a higher risk for elevated trans-prosthetic gradients and prosthesis-patient mismatch in patients with a small aortic annulus. We aimed to assess the short-term hemodynamic performance of self-expanding transcatheter aortic valves with an intra-annular leaflet position in patients with small aortic anatomies. *Materials and Methods*: Consecutive patients with small aortic annuli (annular area < 430 mm^2^), who underwent transcatheter aortic valve implantation with a self-expanding Portico or Navitor (Abbott Medical, St. Paul, MN, USA) transcatheter aortic valve between October 2017 and August 2024 at the University Medical Centre Ljubljana, Slovenia, were analyzed. The main endpoints were the post-procedural mean trans-prosthetic gradient, the presence of moderate or severe prosthesis-patient mismatch or paravalvular regurgitation. *Results*: Overall, 37 patients were included in the study (29 patients with a native aortic valve and 8 patients undergoing valve-in-valve transcatheter aortic valve implantation). The mean age was 81.6 ± 4.3 years, 32 patients (86.5%) were female. The median annular perimeter was 70.8 mm (interquartile range 67.3–74.1 mm) and the median annular area was 379 mm^2^ (interquartile range 355–412 mm^2^). The post-procedural mean trans-prosthetic gradient was 9.0 ± 3.5 mmHg, with no cases with a mean gradient > 20 mmHg. Moderate and severe prosthesis-patient mismatch was observed in 21.2% and 3.0% of patients, respectively. Mild paravalvular regurgitation was noted in 44.1% of patients, there were no cases of moderate or severe paravalvular regurgitation. One patient (3.0%) had moderate valvular regurgitation. *Conclusions*: Self-expanding transcatheter aortic valves with an intra-annular leaflet position are associated with favorable hemodynamic performance in patients with a small aortic annulus.

## 1. Introduction

Transcatheter aortic valve implantation (TAVI) is recommended for the treatment of severe aortic stenosis in the elderly and in those with unacceptably high surgical risk [1]. TAVI is particularly challenging in patients with small aortic annuli as they have a higher risk for prosthesis-patient mismatch (PPM), which is associated with worse outcomes if severe [2]. Transcatheter aortic valves (TAVs) can be either self-expanding or balloon-expandable, and the leaflet position can be supra- or intra-annular. Self-expanding TAVs have been associated with lower post-procedural mean trans-prosthetic gradients and lower rates of PPM than balloon-expandable TAVs in patients with small aortic annuli [3,4]. The recently published SMART randomized trial reported on the outcomes of patients with an annular area ≤ 430 mm^2^, who underwent TAVI with either the self-expanding supra-annular Evolut PRO/PRO+/FX (Medtronic) or the balloon-expandable intra-annular SAPIEN 3/3 Ultra (Edwards Lifesciences) platforms [4]. Although there were no significant differences in clinical outcomes, the trial demonstrated that the supra-annular self-expanding Evolut platform was superior to the balloon-expandable intra-annular SAPIEN platform in terms of bioprosthetic valve dysfunction [4]. The self-expanding but intra-annular Portico/Navitor (Abbott) TAVs were not included in the trial [4]. The aim of this study was therefore to assess the short-term hemodynamic performance of the self-expanding Portico/Navitor TAV, which has an intra-annular leaflet position, in patients with small aortic anatomies.

## 2. Materials and Methods

Consecutive patients, who underwent TAVI with a self-expanding intra-annular Portico or Navitor TAV (Abbott Medical, St. Paul, MN, USA) between October 2017 and August 2024 at the University Medical Centre Ljubljana, Slovenia, were retrospectively analyzed. The inclusion criterion was the presence of a small aortic annulus, defined by an annular area < 430 mm^2^. All patients, who met the criterion were included in the analysis, including patients undergoing valve-in-valve (VIV) TAVI. There were no additional exclusion criteria.

Pre-procedural screening was performed according to local protocol. It included medical history, clinical examination, electrocardiogram, surgical risk assessment, biochemical analysis, pulmonary function tests, echocardiography, carotid arterial doppler examination, coronary angiography, multi-slice computer tomography (MSCT) and dental examination. Patients were evaluated by the institutional multidisciplinary Heart Team, which confirmed the indication for TAVI. The TAV size and type were suggested by the Heart Team, but ultimate selection was left to the interventional cardiologist performing the procedure. As was the decision of performing balloon pre- or post-dilatation. Annular dimensions were determined by MSCT imaging. Patients underwent pre-discharge echocardiography for TAV hemodynamics assessment.

Baseline patient characteristics, pre-procedural screening results, procedural details and pre-discharge echocardiography results were collected prospectively in a dedicated institutional TAVI registry and were later retrospectively crosschecked with available hospital medical records. The study was performed with the approval of The National Medical Ethics Committee of the Republic of Slovenia (No. 0120-315/2024-2711-3, approval date 23 July 2024). Informed consent was not required since this was a registry-based study.

The main endpoints of the study were the post-procedural mean trans-prosthetic gradient, the presence of moderate or severe PPM and paravalvular regurgitation (PVR). Other endpoints of interest were the presence of a mean trans-prosthetic gradient > 20 mmHg, survival to hospital discharge and the rate of new permanent pacemaker implantation (PPI). Patients were considered to have PPM if the indexed effective orifice area (EOAi) was ≤0.85 cm^2^/m^2^ in the case of a body mass index (BMI) < 30 kg/m^2^ or EOAi ≤ 0.70 cm^2^/m^2^ in case of a BMI ≥ 30 kg/m^2^ [5]. PPM was further classified as moderate (EOAi 0.66–0.85 cm^2^/m^2^ for BMI < 30 kg/m^2^ or EOAi 0.56–0.70 cm^2^/m^2^ for BMI ≥ 30 kg/m^2^) and severe (EOAi ≤ 0.65 cm^2^/m^2^ for BMI < 30 kg/m^2^ or EOAi ≤ 0.55 cm^2^/m^2^ for BMI ≥ 30 kg/m^2^), in accordance with the Valve Academic Research Consortium 3 definitions [5]. EOAi was calculated by the continuity equation and indexed to body surface area. PVR was defined according to the three-class grading scheme [5] as absent/trace, mild, moderate or severe by individual cardiologists performing echocardiography.

Patient characteristics were assessed with descriptive statistics. Normally distributed continuous variables are presented with mean and standard deviation and were compared with the Student’s t-test for independent samples. The Kruskal-Wallis test was used to compare continuous variables in case of more than 2 independent groups. Non-normally distributed continuous variables are presented with median and interquartile range (IQR) and were compared with the Mann-Whitney test. The Mann-Whitney test was also used in the case of unequal variances as determined with the Brown-Forsythe test. Normality of data distribution was assessed with the Shapiro-Wilk test. Categorical and nominal variables are presented with number and percentage and were compared with the Fisher’s exact test in case of 2 × 2 contingency tables and with the Freeman-Halton extension of the Fisher’s exact test in case of 3 × 2 contingency tables. Correlation between post-procedural mean trans-prosthetic gradient and EOAi was assessed with the Pearson’s r correlation coefficient. A *p*-value < 0.05 was considered statistically significant. Statistical analysis was performed in JASP (JASP Team (2024), JASP (Version 0.19.1) [Apple Silicon], Amsterdam, The Netherlands).

Baseline patient characteristics and survival to hospital discharge were assessed for all patients with an attempted Portico or Navitor TAV implantation. Post-procedural mean trans-prosthetic gradients, PPM, PVR and the need for new PPI were assessed only for patients following a successful Portico or Navitor TAV implantation.

## 3. Results

A total of 37 consecutive patients with an aortic annular area < 430 mm^2^ underwent TAVI with a self-expanding Portico or Navitor TAV between October 2017 and August 2024 at the University Medical Centre Ljubljana, Slovenia—a high-volume TAVI centre. The mean age was 81.6 ± 4.3 years, 32 patients (86.5%) were female. The median annular perimeter was 70.8 mm (IQR 67.3–74.1 mm) and the median annular area was 379 mm^2^ (IQR 355–412 mm^2^). The pre-procedural echocardiographic characteristics were: mean aortic valve maximal velocity 4.1 ± 0.6 m/s, mean aortic valve gradient 43.5 ± 13.4 mmHg, median aortic valve area 0.6 cm^2^ (IQR 0.5–0.8 cm^2^) and mean left ventricular ejection fraction (EF) 61 ± 11%. Overall, the median surgical risk as determined by Euroscore II calculation was 3.3% (IQR 2.3–6.3%), but it was expectedly higher in patients undergoing VIV TAVI, who had a median Euroscore II of 12.5% (IQR 7.1–17.9%; *p* < 0.001). The Portico TAV was implanted in 22 patients (59.5%) and the Navitor TAV in 15 patients (40.5%). The sizes of selected TAVs were 23 mm, 25 mm and 27 mm, which were implanted in 9 (24.3%), 14 (37.8%) and 14 (37.8%) patients, respectively. Balloon pre-dilatation was performed in 31 patients (83.8%) and post-dilatation in 13 patients (35.1%). Native-valve TAVI was performed in 29 patients (78.4%), whereas 8 patients (21.6%) underwent VIV TAVI. Two patients (5.4%) experienced TAV embolization shortly after deployment. This was in one patient managed with the implantation of a second balloon-expandable TAV, whereas the other patient was converted to surgical aortic valve replacement.

Regarding the outcomes of interest in the remaining 35 patients with a successfully implanted self-expanding intra-annular TAV, the post-procedural mean trans-prosthetic gradient was 9.0 ± 3.5 mmHg, with no patients having a mean trans-prosthetic gradient >20 mmHg. The mean post-procedural doppler velocity index (DVI) was 0.60 ± 0.14 and the mean EOAi was 0.99 ± 0.26 cm^2^/m^2^ (Table 1). Overall, there was a negative correlation between EOAi and the post-procedural mean trans-prosthetic gradient (Pearson’s r −0.645, *p* < 0.001). Most patients had no PPM. Moderate PPM was observed in 7 patients (21.2%) and severe PPM in 1 patient (3.0%). The latter underwent VIV TAVI. Mild PVR was noted in 15 patients (44.1%), there were no cases of moderate or severe PVR. There were no significant differences in the rates of PVR with respect to whether post-dilatation was performed (no post-dilatation: no PVR 50.0%, mild PVR 50.0%, vs. post-dilatation: no PVR 66.7%, mild PVR 33.3%, *p* = 0.476). One patient (3.0%) had moderate valvular regurgitation after native-valve TAVI due to a dysfunctional right coronary cusp. Two patients (5.7%) required new PPI. One patient (2.7%) died following TAV embolization. The remaining 36 patients (97.3%) survived to hospital discharge.

**Figure 1:**
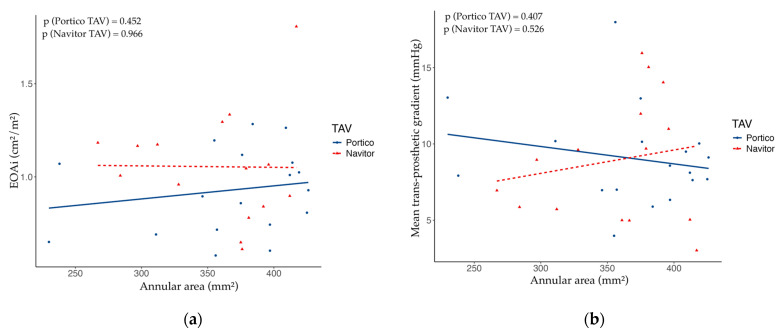
Scatter plots showing the relationship between: (**a**) pre-procedural aortic annular area and post-procedural indexed effective orifice area (EOAi) with respect to implanted transcatheter aortic valve (TAV); (**b**) pre-procedural aortic annular area and post-procedural mean trans-prosthetic gradient with respect to implanted TAV. The indicated *p*-values were calculated with a linear regression model with EOAi and mean trans-prosthetic gradient as dependent variables, and annular area as covariate. In both cases there was no significant correlation between pre-procedural annular area and post-procedural EOAi or mean trans-prosthetic gradient.

With respect to the type of implanted TAV—either Portico or Navitor—there were no significant differences in the post-procedural mean trans-prosthetic gradient (Portico: median 8.5 mmHg, IQR 7.3–10.0 mmHg, vs. Navitor: median 9.0 mmHg, IQR 5.5–11.5 mmHg, *p* = 0.856) or in the incidence of PPM (Portico: no PPM 72.2%, moderate PPM 27.8%, severe PPM 0%, vs. Navitor: no PPM 80.0%, moderate PPM 13.3%, severe PPM 6.7%; *p* = 0.413) (Table 2). Conversely, Navitor TAVs were associated with a lower incidence of mild PVR (Portico: no PVR 36.8%, mild PVR 63.2%, vs. Navitor: no PVR 80.0%, mild PVR 20.0%; *p* = 0.017). The Figure 1 represents the relationship between pre-procedural aortic annular area and post-procedural EOAi or mean trans-prosthetic gradient with respect to the implanted TAV.

When comparing VIV with native-valve TAVI, VIV TAVI was associated with a higher post-procedural mean trans-prosthetic gradient (VIV TAVI 12.0 ± 4.0 mmHg vs. native-valve TAVI 8.0 ± 2.8 mmHg, *p* = 0.004). Conversely, there were no significant differences in the rates of PPM (VIV TAVI: no PPM 62.5%, moderate PPM 25.0%, severe PPM 12.5%; vs. native-valve TAVI: no PPM 80.0%, moderate PPM 20.0%, severe PPM 0%; *p* = 0.267) or PVR (VIV TAVI: no PVR 75.0%, mild PVR 25.0%; vs. native-valve TAVI: no PVR 50.0%, mild PVR 50.0%; *p* = 0.257).

## 4. Discussion

The objective of this study was to assess the valve performance of self-expanding TAVs with an intra-annular leaflet position in patients with a small aortic annulus. The main finding of the study is that self-expanding Portico/Navitor TAVs are associated with a favorable hemodynamic profile in patients with small aortic annuli.

Although individuals with a small aortic anatomy represent a significant proportion of patients undergoing TAVI [6], few prospective clinical trials have evaluated the outcomes of different TAV designs in this subgroup of patients. The randomized CHOICE trial compared the second-generation self-expanding CoreValve (Medtronic) with the balloon-expandable SAPIEN XT (Edwards Lifesciences) TAV in patients with large and small aortic annuli (defined by an annular diameter ≤ 23 mm) [7]. In patients with small aortic annuli, the self-expanding CoreValve was associated with a significantly lower post-procedural mean trans-prosthetic gradient at 30 days (CoreValve 6.7 ± 3.2 mmHg vs. SAPIEN XT 10.2 ± 4.2 mmHg, *p* < 0.001). There were no significant differences in the rates of PPM (CoreValve 15.4% vs. SAPIEN XT 23.3%, *p* = 0.517) or aortic regurgitation (CoreValve: mild 56.7%, moderate 0%; SAPIEN XT: mild 40.0%, moderate 5.7%, *p* = 0.218) [7]. On the other hand, the non-randomized CHIOCE-Extend registry compared the third-generation self-expanding Evolut R (Medtronic) with the balloon-expandable SAPIEN 3 (Edwards Lifesciences) TAV [7]. Implantation of the self-expanding Evolut R resulted in lower 30-day mean trans-valvular gradients (Evolut R: 6.6 ± 3.1 mmHg vs. SAPIEN 3: 13.3 ± 4.3 mmHg, *p* < 0.001) and lower rates of PPM (Evolut R: 33.3% vs. SAPIEN 3: 59.2%, *p* = 0.029) in comparison with the balloon-expandable SAPIEN 3. There were no significant differences in the incidence of aortic regurgitation (Evolut R: mild 47.1%, moderate 0%; SAPIEN 3: mild 43.8%, moderate 0%, *p* = 0.836) [7].

The much more recent SMART randomized trial reported on the outcomes of the self-expanding Evolut R/PRO/PRO+/FX (Medtronic) in comparison with the balloon-expandable SAPIEN 3/3 Ultra (Edwards Lifesciences) TAVs in patients with an annular area ≤430 mm^2^ [4]. There were no significant differences with respect to 12-month all-cause mortality, disabling stroke or hospital readmission for heart failure. However, self-expanding TAVs were associated with lower 12-month mean trans-prosthetic gradients (self-expanding TAVs: 7.7 ± 4.0 mmHg vs. balloon-expandable TAVs: 15.7 ± 6.7 mmHg, *p* < 0.001) and lower rates of moderate or severe PPM at 30 days (self-expanding TAVs: 10.3% vs. balloon-expandable TAVs: 35.1%, *p* < 0.001) [4]. By comparison, both the reported mean trans-prosthetic gradient and rate of moderate or severe PPM in the self-expanding Evolut group were lower than we observed in our study. However, direct comparisons are difficult, since we also included patients following VIV TAVI.

Regarding the Portico/Navitor TAVs (Abbott), registry based studies of patients with small aortic annuli have reported mean trans-prosthetic gradients ranging from 8.9 ± 0.3 mmHg to 9.2 ± 4.5 mmHg, with rates of more than mild PVR from 19.0% to 19.2%, moderate PPM from 20.5% to 38.7% and severe PPM from 9.0 to 14.0% [3,8]. The mean trans-prosthetic gradients and rate of moderate PPM were comparable to those observed in our study. On the other hand, we noted numerically lower rates of severe PPM and no cases of moderate or severe PVR. Although a substantial proportion of patients in our study had mild PVR, the rate was comparable to those of other self-expanding supra-annular and balloon-expanding intra-annular platforms in the CHOICE and CHOICE-Extend studies [7]. Conversely, the TAVI-SMALL and TAVI-SMALL 2 registry studies reported only more than mild PVR for the Portico platform, so a direct comparison of the rates of mild PVR in not possible [3,8]. Our approach to minimizing the rates of PVR is by performing balloon pre-dilatation, and post-dilatation in cases of significant PVR on angiography following TAV deployment. However, due to the small sample size and consequently low statistical power, we were not able to demonstrate a significant reduction in PVR following balloon post-dilatation. It is notable, that we observed also a lower need for new PPI than comparable observational studies [3,8]. This may be in part explained by the fact that a large proportion of patients in our study underwent VIV TAVI, which is associated with a lower risk for new PPI [9]. In fact, in our case no patient required new PPI following VIV TAVI. Additionally, to reduce the risk for PPI we try to avoid low TAV positioning and oversizing.

Our study also included patients undergoing VIV TAVI. In general, such VIV TAVI procedures are associated with low mortality and heart failure readmissions, but also with higher post-procedural mean trans-prosthetic gradients in comparison with native-valve TAVI [10]. A study of 56 consecutive patients following VIV TAVI with the Portico TAV reported a post-procedural mean trans-prosthetic gradient of 16.2 ± 9.3 mmHg, with 25% of patients with a mean gradient > 20 mmHg [11]. Conversely, a recently published case series of 5 consecutive patients after VIV TAVI with the latest-generation Navitor system reported a much lower 30-day mean trans-prosthetic gradient of only 6.0 mmHg [12].

In this study two patients experienced TAV embolization in the ascending aorta during a native-valve TAVI procedure. In the first patient TAV embolization occurred at the time of TAV disconnection from the delivery system. Implantation of a second balloon-expandable TAV was attempted but was not successful. The patient was therefore converted to surgical aortic valve replacement and TAV removal, which was successfully performed. In the second patient embolization occurred due to high TAV positioning during deployment. It caused an acute obstruction of the coronary ostia leading to cardiac arrest. The patient was placed on mechanical circulatory support with extracorporeal membrane oxygenation, followed by redo TAVI with a second balloon-expandable valve. The patient ultimately died due to progressive respiratory failure. In general, TAV embolization and migration is a rare complication, occurring in less than 1% of TAVI cases, but is associated with increased morbidity and mortality [13]. Self-expanding platforms and first-generation devices have been associated with an increased risk for embolization [13]. On the other hand, preventable causes include device malposition and manipulation as well as inadequate sizing, underscoring the need for proper valve size selection and visualization during deployment [13].

Finally, this study has some inherent limitations that need to be addressed. First, we must acknowledge the small sample size with a total of only 37 subjects. This may have reduced the statistical power of the study for detecting significant differences in subgroup analyses. Although there were numerical differences in the post-procedural rates of PPM and PVR in subgroup analyses, these were mostly not statistically significant. Nevertheless, we were still able to demonstrate an association between VIV TAVI and higher trans-prosthetic gradients, and a lower incidence of PVR with the use of Navitor system.

Although this study presents the outcomes of an all-comer patient population, intra-annular self-expanding TAVs were implanted in a relatively small number of carefully selected patients, increasing the risk for patient selection bias. In combination with the small sample size this limits the generalizability of the results to the whole population of patients undergoing TAVI in small aortic annuli. Additionally, since data were retrieved from accessible medical records, there is the possibility of underreporting. Pre- and post-procedural echocardiography was not performed by a dedicated core laboratory, which may have led to interobserver variability, particularly when assessing the grade of PVR. Nevertheless, we believe this study gives insight into the hemodynamic performance of self-expanding TAVs with intra-annular leaflets in an all-comer population of carefully selected patients with small aortic anatomies, including patients undergoing VIV TAVI, who were often excluded from previous trials.

## 5. Conclusions

TAVI with self-expanding TAVs with an intra-annular leaflet position is associated with a favorable hemodynamic profile in patients with small aortic anatomies. Larger studies are needed to confirm our results.

## Figures and Tables

**Table 1:** EOAi overall and with respect to TAV type, size and TAVI procedure.

	EOAi (cm^2^/m^2^)	*p*-Value
Overall	0.99 ± 0.26	-
TAV type		
Portico	0.93 ± 0.22	0.170 *
Navitor	1.06 ± 0.30	
TAV size		
23 mm	1.02 ± 0.19	0.738 ^#^
25 mm	0.94 ± 0.25	
27 mm	1.03 ± 0.33	
TAVI procedure		
Native-valve	1.02 ± 0.26	0.205 *
VIV	0.88 ± 0.23	

* Student’s *t*-test for independent samples; ^#^ Kruskal-Wallis test; EOAi—indexed effective orifice area; TAV—transcatheter aortic valve; TAVI—transcatheter aortic valve implantation; VIV—valve-in-valve.

**Table 2:** Post-procedural outcomes with respect to transcatheter aortic valve type.

	Transcatheter Aortic Valve Type		
	Portico	Navitor	*p*-Value
Mean gradient (mmHg) *	8.5 (7.3–10.0)	9.0 (5.5–11.5)	0.856
EOAi (cm^2^/m^2^) ^#^	0.93 ± 0.22	1.06 ± 0.30	0.170
PVR ^§^			
No/trace	36.8%	80.0%	0.017
Mild	63.2%	20.0%	
>mild	0	0	
New PPI ^§^	0	13.3%	0.176

* median (interquartile range); ^#^ mean ± standard deviation; ^§^ percent. EOAi—indexed effective orifice area; PPI—permanent pacemaker implantation; PVR—paravalvular regurgitation.

## Data Availability

Available upon request from the corresponding author.
